# Big effects of small RNAs on legume root biotic interactions

**DOI:** 10.1186/s13059-014-0475-2

**Published:** 2014-09-24

**Authors:** Huan Wang, Nam-Hai Chua

**Affiliations:** Laboratory of Plant Molecular Biology, Rockefeller University, New York, NY 10021 USA

## Abstract

Comprehensive profiling of microRNAs (miRNAs) from the legume *Medicago truncatula* reveals the organization of miRNA-based regulatory modules in root biotic interactions.

## Role of small RNAs and biotic stress responses of plants

Recent research has uncovered a large number of genomic loci encoding long or small RNAs with low protein-coding potential. Molecular and *in silico* studies of such non-coding RNAs (ncRNAs) have expanded our understanding of gene transcriptional regulation and the generation of organism complexity. In plants, the most well studied ncRNAs are 21 to 24 nucleotide small RNAs (smRNAs) that negatively regulate specific targets through complementary sequences. Interestingly, plant microRNAs (miRNAs) have been implicated in regulating a wide range of developmental processes and stress responses [1].

Plants generate root systems with complex architectures to acquire water and nutrients and to interact with various soil microorganisms. Among soil microorganisms, arbuscular mycorrhizal (AM) fungi and bacterial *Rhizobium* can promote plant growth as well as control fungal diseases [2], suggesting that there is an interconnection of signaling pathways mediating root symbiotic and pathogenic interactions, and miRNAs are implicated in these biotic relationships. Although previous work on miRNA networks has helped to uncover novel mechanisms involved in plant adaptation to changing environmental conditions [3], how beneficial microbes affect host pathogenic responses remains unclear [4].

In this issue of *Genome Biology*, the groups of Martin Crespi and Laurent Gentzbittel characterize the miRNAome of the leguminous Barrel Medic *Medicago truncatula* root under a variety of symbiotic and pathogenic interactions and in response to related signals [5]. Taking advantage of re-sequencing of the *M. truncatula* genome, they identify many novel miRNAs; in addition, their comprehensive miRNA profiling experiments enable them to further decipher miRNA-based regulation modules in response to specific biotic interactions.

## Conservation of known and novel miRNAs and adaption to environments

Analysis of the phylogenetic relationship of known and novel *M. truncatula* miRNAs among three legumes, three non-legume eudicots and two monocots allowed Formey and colleagues to classify *M. truncatula* miRNAs into three groups: (1) widely conserved; (2) legume specific; and (3) *M. truncatula* specific. Similar to *Arabidopsis* miRNAs, a large number of *M. truncatula* miRNAs that belong to the first group are deeply conserved. Approximately 200 novel miRNAs are conserved in all eight selected angiosperm species, which considerably expands the current miRNA database. Moreover, the authors’ results also suggest that current identification of plant miRNAs is still not yet saturated, even in well-studied species. Additional miRNAs are expected to be discovered by deeper detection methods or in samples derived from specific organs, developmental stages or under certain treatments [6].

The birth and evolution of miRNA genes is an issue of considerable interest as it could potentially help investigators understand how miRNA-based regulation networks adapt to various environments. To investigate the selection of miRNA genes during evolution, Formey *et al.* performed polymorphism analyses in 26 ecotypes of *M. truncatula*. Not surprisingly, they rarely found SNPs at positions 10 and 11 of either known or novel mature miRNAs, which are crucial for the silencing function. Analysis of sequence polymorphisms of pre-miRNA and flanking regions showed that conserved miRNAs usually carry more genetic variations than novel miRNAs, especially on the loop of pre-miRNA and upstream and downstream regions. These results are consistent with a previous model that miRNAs might have been derived from inverted-duplication of targeted protein-coding genes [7]. Formey and colleagues also proposed a balancing selection in miRNA evolution and adaptation to different environments and have provided a rich data-set for future analysis of miRNA evolution.

## The structure of miRNA-based regulation modules in response to pathogenic interactions, symbiotic interactions and signals

Symbiotic signals are essential for the initial steps of interactions between legumes and both AM fungi and the root-nodule bacterium *Rhizobium*. Comparing miRNA abundance in roots treated with or without purified non-sulfated Myc-LCO and Nod factors, Formey *et al.* found that miRNAs responsive to the two signal triggers - Myc-LCO and Nod factors - are largely different. These differentially regulated miRNAs, which include both known and novel species, serve as good candidates for further analysis of how the two symbiotic interactions become specialized.

By contrast, AM fungi and *Rhizobium* also play an important role in influencing plant disease resistance as they are known to reduce pathogen-elicited damage in host plants. Many factors can account for this reduced damage - for example, improved nutrient status of the host plant, change in root growth and morphology, competition for colonization sites and host photosynthates, changes of microbial community composition and activation of plant defense mechanisms [2]. Recent studies reveal that miRNAs might also be involved in regulating plant defense responses during symbiotic interactions [4]. However, it is still unclear how miRNA-based regulation networks modulate common and specific responses to pathogenic and symbiotic interactions. To obtain an overview of miRNAs involved in root interaction with pathogenic microorganisms and/or symbionts, Formey and colleagues compared the miRNA population of inoculated roots with that of mock-inoculated controls. Interestingly, miRNAs regulated by all pathogens and symbionts are mostly known miRNAs, whereas the majority of novel miRNAs are preferentially responsive to specific microorganisms. These miRNAome data are clearly a rich resource for further investigations on pathogenic and symbiotic pathways.

Finally, Formey *et al.* applied weighted gene coexpression network analysis (WGCNA) to excavate miRNA regulatory modules that could commonly or specifically respond to biotic interactions and symbiotic signals. Each module contains both known and newly identified miRNAs. Moreover, functional enrichment analysis of the predicted targets of miRNAs in each module suggests that each module could be involved in regulating significantly different biological processes. Their modules suggest several testable hypotheses on the function of miRNAs in discriminating between different root biotic interactions.

## Concluding remarks

Formey and colleagues present comprehensive identification and profiling of many known and novel miRNAs in response to pathogenic and symbiotic interactions and signals in a model legume, *M. truncatula.* Their phylogenetic analysis uncovers many deeply conserved novel miRNAs. However, there are clear differences between different legumes regarding the functions of some symbiosis-related miRNAs. For example, miR397 is induced by root pathogenic and symbiotic interactions in soybean, but this miRNA is absent from *M. truncatula* [8]. Therefore, it will be also be interesting to study the birth of new miRNAs and expansion of known miRNA families in legumes and investigate how new miRNA (members) gain novel and specific functions in nodulation.

Another interesting topic addressed by Formey *et al.* is the role of miRNA-based regulation modules in defining commonalities and differences between root pathogenic and symbiotic interactions. Considering the overlap between these two biotic interactions, it is important to investigate regulation of miRNAs at the initial signaling steps to capture early events. Thus, further studies might include pathogenic signal triggers, such as flagellin and chitin, and their responses compared with those obtained from symbiotic signals. Understanding the molecular basis of root biotic interactions will help us to modify pathogenic and symbiotic relationships for the benefit of sustainable agriculture. In this regard, the exciting results described by Formey *et al.* provide a rich data-set and useful information for further analysis of legume miRNAs in response to root biotic interactions.

## Abbreviations

AM: Arbuscular mycorrhizal
LCO: Lipochitooligosaccharides
ncRNA: Non-coding RNA
smRNA: Small RNA
WGCNA: Weighted gene coexpression network analysis
